# Impact of a first psychosis program in clinical variables after two years of follow-up

**DOI:** 10.1192/j.eurpsy.2021.1411

**Published:** 2021-08-13

**Authors:** M. Martinez, E. García De Jalón, N. Pereda, A. Fernández, M.C. Ariz, L. Azcárate, M. Otero

**Affiliations:** Salud Mental. Primeros Episodios, Servicio Navarro de Salud, Pamplona, Spain

**Keywords:** psychosis, early intervention, schizophrénia, First Psychosis Program

## Abstract

**Introduction:**

Early Intervention Services for Early-Phase Psychosis have shown efficacy and effectiveness (Correl C, JAMA). In Pamplona, Spain, there is an Early Intervention Program that has been providing multiprofesional assistance for First Psychotic Patients for the last two years.

**Objectives:**

The aim of this study is to analize the longitudinal effects of the different interventions in several clinical variables applied to 240 patients during two years of follow-up : CASH dimensions, substance abuse, antipsychotic type and dosage, remission rates, re-hospitalization rates and DSM 5 diagnoses.

**Methods:**

We apply an standard evaluation protocol to every patient at different times: premorbid, initial time and at months 6, 12, 18 and 24. We analyse the data with the SPSS statistical program to see the results in these variables.

**Results:**

The positive and disorganized dimensions show an evident decline during the treatment. The doses of antipsychotic drugs are low and tend to decline. 87% of patients are in monotherapy. The most frequent DSM 5 basal diagnosis is Brief Psychotic Episode, but during de follow-up the Diagnosis of Schizophrenia increase from 14,6% at baseline up to 46,2% at month 24. The remission rates are about 65% after 24 months.

**Conclusions:**

Early Intervention Services improve psychopathological dimensions, prevents from re-hospitalization, allows the use of lower doses of Antipsychotic Drugs and improve the rates of remission. However, the diagnosis of Schizophrenia is high, so there is no evidence that these programs prevents from chronicity, but provide a better quality of life.

